# Diagnostic Accuracy of the Screening Questionnaires for Obstructive Sleep Apnoea in Pregnancy: A Meta‐Analysis and Updated Systematic Review

**DOI:** 10.1111/jsr.70197

**Published:** 2025-09-25

**Authors:** Marco La Verde, Esposito Renata, Maria Maddalena Marrapodi, Luigi Della Corte, Mario Fordellone, Hande Uzunçıbuk, Vincenzo Ronsivalle, Gabriele Cervino, Giuseppe Minervini

**Affiliations:** ^1^ Department of Woman, Child and General and Specialized Surgery University of Campania “Luigi Vanvitelli” Naples Italy; ^2^ Department of Environmental Biological and Pharmaceutical Sciences and Technologies University of Campania “Luigi Vanvitelli” Caserta Italy; ^3^ Department of Woman, Child and General and Specialized Surgery, Pediatric Unit University of Campania Luigi Vanvitelli Naples Italy; ^4^ Saveetha Dental College and Hospitals, Saveetha Institute of Medical and Technical Sciences (SIMATS) Saveetha University Chennai India; ^5^ Department of Neuroscience, Reproductive Sciences and Dentistry, School of Medicine University of Naples Federico II Naples Italy; ^6^ Medical Statistics Unit University of Campania Luigi Vanvitelli Naples Italy; ^7^ Department of Orthodontics, Faculty of Dentistry Trakya University Edirne Turkiye; ^8^ Department of Biomedical and Surgical and Biomedical Sciences Catania University Catania Italy; ^9^ Department of Biomedical and Dental Sciences, Morphological and Functional Images University of Messina Messina Italy; ^10^ Multidisciplinary Department of Medical‐Surgical and Odontostomatological Specialties University of Campania “Luigi Vanvitelli” Naples Italy

**Keywords:** apnoea hypopnea index, Berlin questionnaire, Epworth sleepiness scale, home sleep apnoea test, obstructive sleep apnoea, pregnancy

## Abstract

This systematic review and meta‐analysis evaluate the diagnostic performance of obstructive sleep apnoea (OSA) screening tools in pregnant populations and the efficacy of common sleep questionnaires. A comprehensive search was conducted from the beginning to March 2024 using MEDLINE, Scopus, Cochrane CENTRAL, and Google Scholar, adhering to PRISMA guidelines. Studies were included if they adopted OSA screening questionnaires in pregnant women and compared results with overnight polysomnography. Keywords included terms related to pregnancy and OSA (e.g., Berlin questionnaire, ESS, PSQI, PSG, Watch‐PAT). Sensitivity, specificity, diagnostic odds ratios (DOR), and likelihood ratios (LR) were calculated for each study. Tests for equality of sensitivity and specificity were conducted to evaluate variability. Eight studies involving 10,043 pregnant women were included. Reported OSA prevalence ranging from 12% to 72.3%. The Berlin questionnaire demonstrated significant heterogeneity in both sensitivity (*χ* ^2^ = 23.54, df = 6, *p* = 0.0006) and specificity (*χ* ^2^ = 33.74, df = 6, *p* = 7.56e−06), with a strong positive correlation between sensitivity and false positive rate (max. correlation coefficient = 0.994; 95% CI 0.092–0.994). The ESS showed similar variability (sensitivity: *χ* ^2^ = 10.55, df = 4, *p* = 0.0321; specificity: *χ* ^2^ = 74.18, df = 4, *p* = 2.97e−15), also revealing a positive correlation between sensitivity and false positives. Traditional screening tools such as the Berlin questionnaire and ESS exhibit poor diagnostic accuracy for OSA during pregnancy. These findings highlight the need for pregnancy‐specific screening instruments and further research into OSA risk factors unique to this population.

## 1 Background

Obstructive sleep apnoea (OSA) in pregnancy represents a common and often underdiagnosed disease (Liu et al. 2019). Any physiological changes of pregnancy impact normal respiration and sleep and, in patients with other risk factors, improve OSA development (Venkata and Venkateshiah 2009). For example, the associated gestational physiological and hormonal changes modify the sleep architecture and worsen breathing disorders (Soma‐Pillay et al. 2016). These changes modify the pregnant oxygen levels and increase the rate of hypertensive disorders in pregnancy or foetal growth restriction (Venkata and Venkateshiah 2009). Recent studies indicate that OSA may affect between 12% and over 70% of pregnant women (Venkata and Venkateshiah 2009).

Understanding these variations becomes indispensable not just for the diagnosis and treatment per se of sleep disorders but also for the prediction and control of negative maternal and foetal outcomes related to OSA impacts (Izci‐Balserak and Pien 2010). Emerging evidence focused on the effect of oxygen saturation during OSA on pregnancy outcomes suggests that successful screening and management methods can improve the outcomes (Dominguez, Krystal, et al. 2018). Several articles explore the association of OSA in pregnant women with the following gestational complications: pre‐eclampsia, gestational hypertension and diabetes (Liu et al. 2019; Bourjeily et al. 2017). Early screening represents a potential tool to improve both maternal and foetal outcomes (Silvestri and Aricò 2019; La Verde et al. 2022). Different screenings were applied over time to perform OSA screening in pregnant women. Whereas the OSA prevalence is generally high, the reporting of diagnostic accuracy varies between the different screening methods used by different authors across the literature. The questionnaires were applied as screening tools for OSA identification in the pregnant cohort, but this area remains critically under‐researched. Most notably, there is a wide variation in sensitivity and specificity across varied populations and clinical settings for the varied screening tools, and their universal applicability and effectiveness remain controversial. The Berlin questionnaire consists of 10 self‐administered questions divided into 3 categories: snoring component, daytime sleepiness, and obesity or chronic hypertension. Positive for two out of three categories is considered a high risk for OSA (Netzer et al. 1999). The ESS measures daytime sleepiness with eight questions concerning the doze‐off. The ESS total scores are 0–24, and a cut‐off of ≥ 10 represents a high risk for OSA (Johns 1991). A meta‐analysis analysed the questionnaire's accuracy in pregnancy and suggested a poor performance during pregnancy (Tantrakul et al. 2017). The clinical question remains: what is the most sensitive, specific, cost‐effective, and practical tool for early identification of OSA in pregnant women? In summary, the objective of the present systematic review and meta‐analysis study is to define the diagnostic accuracy of several screening questionnaires for this important gap identified in the screening of OSA during pregnancy. The review was designed to synthesise and analyse novel findings and to give a clear view of efficacy regarding the use of these tools within a clinical setting.

## 2 Materials and Methods

This review was conducted according to the recommendations from the preferred reporting items for systematic review and meta‐analysis (Liberati et al. 2009) and the methods outlined in Mbuagbaw et al. (2017). The PRISMA checklist is followed in Appendix 1. This study is registered in the International Prospective Register of Systematic Reviews (PROSPERO). Registration Number: CRD42024532097.

### 2.1 Search Strategy

**FIGURE 1:**
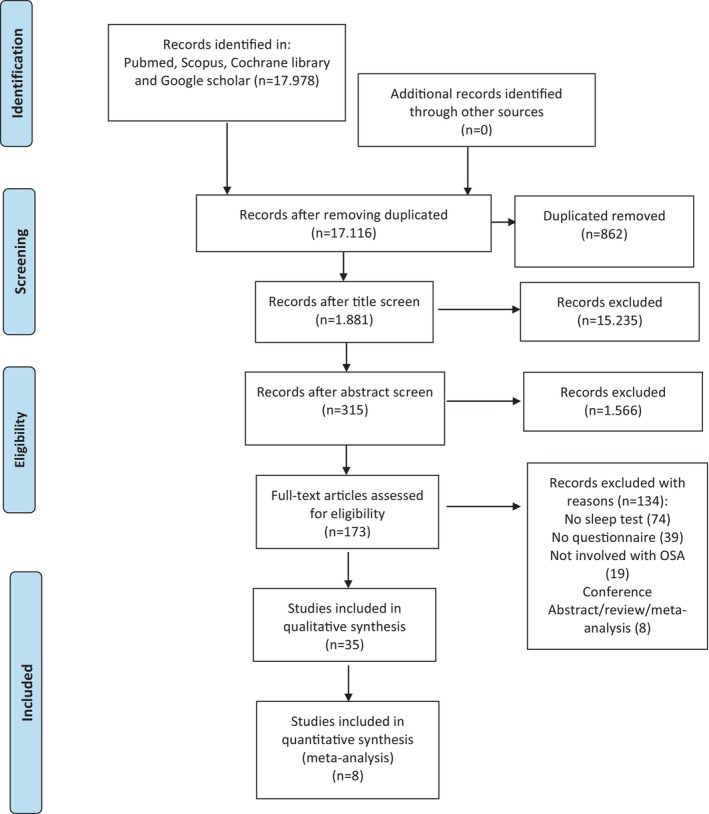
PRISMA flow diagram of the review.

We searched MEDLINE (via PubMed), Scopus, and Cochrane Central Register of Controlled Trials (CENTRAL) from their inception to 1 March 2024 in each of these databases (Figure 1). The search strategy applied text words combined with relevant terminologies regarding the problems as described for the patients, interventions, comparisons, and outcomes, with the following being used as the search keywords: pregnancy, pregnant women, sleep questionnaire, Berlin, Epworth sleepiness scale or ESS, Pittsburgh sleep quality index, PSQI, sleep test, polysomnography, PSG, Watch‐PAT, and OSA, sleep apnoea, obstructive, sleep apnoea, OSA, sleep disordered breathing, SDB (Appendix 2). Grey literature included Google Scholar, and abstracts from international and national conferences were included. We also identified articles from the references of the included papers. There were no language or geographical restrictions. We excluded commentaries, letters to the editor, editorials, reviews, and meta‐analysis from the revision (Figure 1).

### 2.2 Study Selection

#### 2.2.1 Inclusion and Exclusion Criteria

We assessed the studies where the OSA questionnaires were carried out during pregnancy. The research question was formulated according to the PICO framework: “In pregnant women, what is the accuracy of the obstructive sleep apnea questionnaires?” This question tries to clarify the screening of the OSA in pregnancy, pointing to understanding the performance of these tools. The specified PICO question was then: P—population: pregnant women; I—intervention: analyses of positive/negative OSA questionnaires; C—control: negative OSA questionnaires; O—outcome: sensitivity and specificity of the OSA questionnaires as confirmed with overnight polysomnography; S—study designs: all types of analytic studies (cross‐sectional, cohort, case–control, clinical trial) assessing the OSA questionnaires in pregnancy. The two authors (E.R. and M.L.V.) systematically extracted data according to the abstracts that were included in the study. After the complete text review of the study, the first and second authors extracted data independently that related to the detail of the study and independent data that answered the outcome of interest. In cases of disagreement, a third reviewer (L.D.C.) was consulted to reach consensus. Full texts of the selected abstracts were retrieved, and this was part of the data screening procedure which needed title emergence, abstracts, obtaining and reshuffling for rescreening to meet inclusion criteria. Full texts were also taken from titles and abstracts that did not supply the required details on the inclusion criteria. Papers were included when they met the criteria for this review (Figure 1).

### 2.3 Data Extraction

Analysis and graphical renderings of the data were done using Stata version 14.1 (StataCorp, College Station, TX). Data extracted from the included articles for further analysis were demographic information (first authors and year), questionnaire adopted, study‐specific characteristics (study design, patients, pregnancy risk, gestational age), patient characteristics (age and BMI), sleep test/criteria adopted, OSA prevalence (Table 1). Diagnostic data and performance of the questionnaires during pregnancy were extracted and described in Table 2.

**TABLE 1:** Characteristics of the included studies.

Author, year, questionnaire	Study characteristics	Patient characteristics			Diagnosis	OSA	
		Age mean (SD) (y)	GA mean (SD) (wk)	BMI mean (SD) (kg/m^2^)	Sleep test/criteria	Prevalence	Severity
Tantrakul et al. (2015) Berlin ESS	*Study design*: cross sectional study *Patients*: singleton pregnancy N1/472 *Setting*: high risk ANC clinic	33.1 (5.2)	22.8 (9.2)	Pre‐pregnancy 24.2 (5.3)	Watch‐PAT200AHI 5	31.9%	Median (IQR) AHI overall 2.4 (8.0)
	*Pregnancy risk*: high *GA*: any *Country*: Thailand			Pregnancy 26.9 (5.3)	Automated analysis on PAT software algorithm		
Lockhart et al. (2015) STOP ESS	*Study design*: prospective cohort *Patients*: volunteer singleton pregnancy	28 (6.3)	32 (3.1)	Pre‐pregnancy n/a	ApneaLink AHI 5 Apnea 1/4 reduction of airflow to 0e20% lasting 10 s	12%	n/a
ASA‐checklist	*N 1/4 248 setting*: OPD and IPD ANC service *Pregnancy risk*: g*eneral GA*: 27 weeks *Country*: USA			Pregnancy median (IQR) 31(27e36)	Hypopnea 1/4 reduction of airflow to 50% lasting 10 s		
Wilson et al. (2013) Berlin MAP Index	*Study design*: Prospective cohort *Patients*: singleton pregnancy N1/443 *Setting*: ANC clinic *Pregnancy risk*: general	33.5 (5.1)	22.3 (4.0)	Pre‐pregnancy 32.2 (8.0)	PSG RDI5 RDI10 Scoring based on AASM 2007 criteria, alternative hypopnea rule (defined as reduction of nasal pressure signal at least 50% lasting 10 s with either oxygen desaturation 3% or arousal)	35%	Median (IQR) AHI Without OSA: 1.5 (0.6–2.7)
	*GA*: 2nd–3rd trimesters *Country*: Australia			Pregnancy 37.5 (7.9)			With OSA: 6.2 (4.9–13.2)
Fung et al. (2013) Berlin MAP Index	*Study design*: prospective cohort *Patients*: convenient sample of singleton pregnancy N1/441	31.2 (?)	21.4 (2.4)	Pre‐pregnancy 26.1 (6.4)	PSG RDI5 scoring based on AASM 2007 criteria, alternative hypopnea (defined as reduction of nasal pressure signal at least 50% lasting 10 s with either oxygen desaturation 3% or arousal)	34%	Median (IQR) RDI without OSA: 1.4 (0.6–2.6)
	*Setting*: ANC clinic *Pregnancy risk*: General *GA*: 2nd trimester with 3rd trimester follow‐up *Country*: Australia			Pregnancy n/a			With OSA: 6.2 (4.9–11.7)
Facco et al. (2014) Berlin ESS	*Study design*: prospective cohort *Patients*: Singleton pregnancy N1/4100	33.0 (6.5)	16.5 (3.7)	Pre‐pregnancy 31.9 (9.1)	Watch‐PAT100 AHI 5 automated analysis on PAT software algorithm	28%	Median (IQR)AHI overall 1.5 (0.5–6.0)
	*Setting*: ANC clinic *Pregnancy risk*: High risk for OSA *GA*: 6e20 week *Country*: USA			Pregnancy n/a			
Olivarez et al. (2010) Berlin	*Study design*: prospective cohort *Patients*: singleton pregnancy N1/4100	26.6 (7.1)	32.3 (3.5)	Pre‐pregnancy n/a	ApneaLink AHI 5 Apnoea and hypopnea definitions were not specified	20%	n/a
	*Setting*: antepartum obstetric admission *Pregnancy risk*: high *GA*: 26 weeks *Country*: USA			Pregnancy 27.5 (7.2)			
Bajaj et al. 2023 ESS	*Study design*: observational study *Patients*: pregnancy *N* = 159	33.4 (5.6)	21.9 (8.1)	Pre‐pregnancy 40.6 (8.3)	Home sleep study	72.3%	n/a
	*Pregnancy risk*: general *GA*: Any *Country*: Wisconsin‐Madison			Pregnancy n/a	Polysomnography (PSG)		
Dominguez, Grotegut, et al. (2018) Berlin ESS	*Study design*: observational study *Patients*: pregnancy *N* = 80	30.0 (?)	29.8 (?)	50.5 (?)	Type III home sleep apnoea test (ApneaLink Air; ResMed, Poway, CA)	24%	n/a
ASA‐checklist	*Pregnancy risk*: high risk *GA*: Any *Country*: Durham, North Carolina						

**TABLE 2:** Diagnostic data and performance of Berlin questionnaire and Epworth sleepiness scale for screening of OSA during pregnancy.

Questionnaire/study	TP	FP	FN	TN	Sensitivity (95% CI)	Sensitivity (95% CI)
Berlin						
Tantrakul et al. 2015	13	6	10	43	0.57	0.88
Lockhart et al. 2015	22	85	8	133	0.73	0.73
Wilson et al. 2013	13	19	2	9	0.87	0.32
Fung et al. 2013	13	13	1	14	0.93	0.52
Facco et al. (2014)	11	23	17	49	0.39	0.68
Olivarez et al. 2010	7	29	13	51	0.35	0.64
Dominguez, Grotegut, et al. (2018)	2	17	2	59	0.79	0.20
ESS						
Tantrakul et al. 2015	9	19	14	28	0.39	0.60
Lockhart et al. 2015	15	88	11	119	0.58	0.57
Facco et al. (2014)	10	17	18	55	0.36	0.76
Dominguez, Grotegut, et al. (2018)	2	17	3	58	0.00	0.10
Bajaj et al. 2023	30	85	13	26	0.26	0.70

### 2.4 Quality Assessment

Methodological quality of the studies was assessed by two independent reviewers (E.R. and M.L.V.) according to QUADAS‐2 (quality assessment of diagnostic accuracy studies) (Whiting et al. 2011). The QUADAS‐2 tool focuses on the risk of bias (internal validity) and applicability (external validity) of the tool in relation to its domains, including patient selection, index test, reference standard, and flow and timing between index test and reference standard. Each domain is graded as low, high, or unclear risk of bias (Whiting et al. 2011).

### 2.5 Statistical Analysis

A bivariate meta‐analysis with a random‐effects model was applied for pooling diagnostic parameters (i.e., sensitivity, specificity, likelihood ratio (LR) positive/negative, and diagnostic odds ratio (DOR)). To conduct this meta‐analysis, several statistical methods were employed to assess the diagnostic accuracy of the Berlin and ESS questionnaires based on data from the seven primary studies included. Firstly, the sensitivity and specificity of each study, along with 95% confidence intervals, were calculated. Subsequently, tests for equality of sensitivity and specificity among the studies were performed to evaluate result heterogeneity. DOR and LR were also computed to further assess the diagnostic performance of the questionnaires. Lastly, a correlation analysis was conducted to examine the relationship between sensitivity and false positive rates across the included studies. All calculations were carried out with a 95% confidence interval, and a continuity correction of 0.5 was applied, if applicable, to ensure the accuracy and reliability of the results. All the statistical analyses were performed with R Studio statistical software. In view of the variability in reference standards between the included studies (Apnea‐Link, Watch‐PAT, and reference PSG), we applied a random‐effects bivariate model to account for between‐study heterogeneity. Despite methodological variation, pooling of studies was considered appropriate to confirm the OSA diagnosis and provided extractable diagnostic accuracy metrics for the same screening questionnaires.

## 3 Results

### 3.1 Eligible Studies

Out of the 17,978 articles, 862 were duplicates. Therefore, 17,116 were evaluated for title and abstract screening to establish their inclusion eligibility (Figure 1). One thousand eight hundred and eighty‐one studies were assessed at the abstract screen. A final 8 out of 173 screened articles were selected. The quantitative synthesis comprised eight studies (Tantrakul et al. 2015; Lockhart et al. 2015; Wilson et al. 2013; Fung et al. 2013; Facco et al. 2014; Olivarez et al. 2010; Bajaj et al. 2023; Dominguez, Grotegut, et al. 2018). The questionnaires evaluated were: seven studies utilised the Berlin questionnaire, five studies used the ESS, one study used the STOP questionnaire, and one study used the ASA checklist. Given the restricted data, the meta‐analysis was performed for two questionnaires: the Berlin and ESS (Tables 1 and 2).

### 3.2 Study Characteristics of the Included Studies

The studies included were conducted between the years 2010 and 2023. The full list of the studies included, and study characteristics (including year, questionnaire, study design, setting, pregnancy risk, country), patient characteristics (age, gestational age, BMI), sleep test/criteria, and OSA characteristics (prevalence and severity) are described in Table 1. Table 2 shows the diagnostic data and performance of each OSA screening test in pregnant patients. A total of 8 studies with 10,043 subjects were included in this meta‐analysis (Tantrakul et al. 2015; Lockhart et al. 2015; Wilson et al. 2013; Fung et al. 2013; Facco et al. 2014; Olivarez et al. 2010; Bajaj et al. 2023; Dominguez, Grotegut, et al. 2018). Seven studies analysed the performance of the Berlin questionnaire (Tantrakul et al. 2015; Lockhart et al. 2015; Wilson et al. 2013; Fung et al. 2013; Facco et al. 2014; Olivarez et al. 2010; Dominguez, Grotegut, et al. 2018), five studies analysed the ESS performance (Tantrakul et al. 2015; Lockhart et al. 2015; Facco et al. 2014; Bajaj et al. 2023; Dominguez, Grotegut, et al. 2018) (Table 2). Five studies were conducted in North America (Lockhart et al. 2015; Facco et al. 2014; Olivarez et al. 2010; Bajaj et al. 2023; Dominguez, Grotegut, et al. 2018), two studies in Oceania (Wilson et al. 2013; Fung et al. 2013), and one in Asia (Tantrakul et al. 2015). We included with various definitions of apnoea and hypopnea. One study did not report the definitions of apnoea or hypopnea (Olivarez et al. 2010). The OSA prevalence varied from 12% to 72.3%. Four studies reported the OSA severity (Tantrakul et al. 2015; Wilson et al. 2013; Fung et al. 2013; Facco et al. 2014) (Table 2).

### 3.3 Risk of Bias Assessment

**FIGURE 2:**
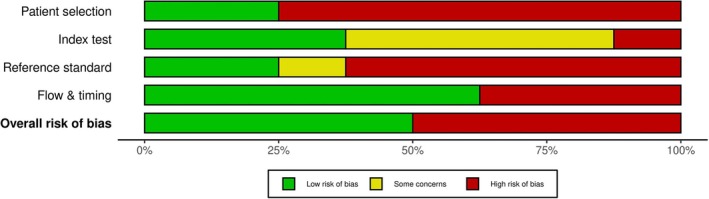
Proportion of risk of bias assessments across included studies using the QUADAS‐2 tool.

Appendix 3 illustrates the quality per study, while Figure 2 summarises the percentage points expressing the quality of methodology across all studies. Considering the patient selection domain, six studies showed a high potential for bias (Tantrakul et al. 2015; Lockhart et al. 2015; Wilson et al. 2013; Fung et al. 2013; Bajaj et al. 2023; Dominguez, Grotegut, et al. 2018). The risk of bias for the index test domain was high only in one study (Lockhart et al. 2015), where there were no pre‐specified criteria for diagnosis, and the results were unclear in four studies (Facco et al. 2014; Olivarez et al. 2010; Bajaj et al. 2023; Dominguez, Grotegut, et al. 2018). With respect to the reference test, the testing in five studies was at high risk for bias because it was performed using Apnea‐Link, ResMed, or Watch‐PAT, rather than standard PSG (Tantrakul et al. 2015; Lockhart et al. 2015; Fung et al. 2013; Facco et al. 2014). One study was unclear regarding the risk of bias, as the information regarding previous knowledge of the test result before study interpretation was unclear (Wilson et al. 2013). The risk of bias assessment in the flow was high risk in three studies (Tantrakul et al. 2015; Bajaj et al. 2023; Dominguez, Grotegut, et al. 2018).

### 3.4 Performance of Berlin Questionnaire

**FIGURE 3:**
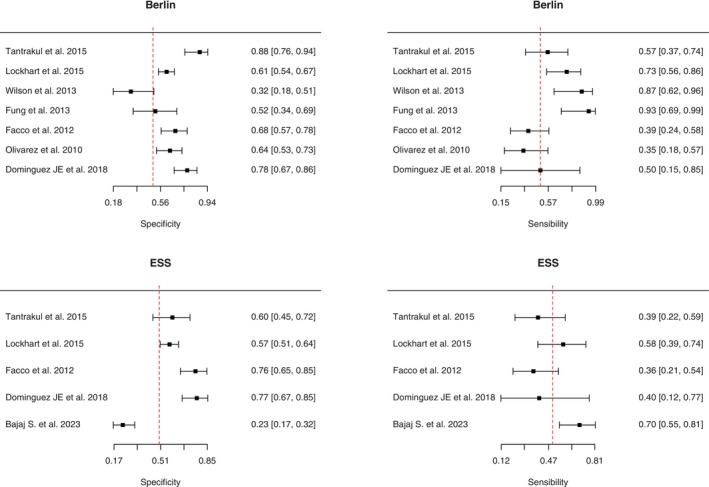
Summary plot of diagnostic accuracy for Berlin questionnaire and Epworth sleepiness scale.

Seven studies adopted the Berlin questionnaire, with a prevalence of OSA that ranges from 12% to 35%. This meta‐analysis investigates the diagnostic accuracy of the Berlin questionnaire based on data from seven primary studies. The findings reveal notable discrepancies in sensitivity and specificity values across the studies. The lowest sensitivity, at 35.0%, is documented in Olivarez et al. (2010), whereas the highest, at 92.9%, is reported in Fung et al. (2013). Similarly, a significant range is observed in specificity, with the lowest value of 32.1% in Wilson et al. (2013) and the highest of 87.8% in Tantrakul et al. (2015). The wide spectrum of results underscores substantial heterogeneity among the included studies. This variability may stem from multiple factors, including differences in participant selection criteria, assessment methodologies for the Berlin questionnaire, and demographic characteristics of the study populations. Moreover, statistical analysis through equality tests highlights significant differences in both sensitivity and specificity among the studies. Regarding DOR and LR, considerable variability is noted among the studies, as reflected in the confidence intervals. The range between minimum and maximum values of DOR, PosLR, and NegLR offers insights into the diversity of diagnostic performances observed across the studies. The test for equality of sensitivities reveals significant differences among the studies (*χ* ^2^ = 23.5398, df = 6, *p* = 0.000634). Similarly, the test for equality of specificities shows statistically significant differences among the studies (*χ* ^2^ = 33.7369, df = 6, *p* = 7.56e−06). Furthermore, a positive correlation between sensitivity and false positive rates, with a maximum correlation coefficient of 0.994 (95% CI 0.092–0.994), suggests that tests with higher sensitivity tend to exhibit higher false positive rates (Figure 3).

### 3.5 Performance of ESS Questionnaire

ESS performance analysis included five studies, Table 2. This meta‐analysis examines the diagnostic accuracy of the Epworth sleepiness scale (ESS) by analysing data from five primary studies. The results reveal significant variation in sensitivity and specificity values among the included studies. The minimum sensitivity recorded is 35.7%, observed in Facco et al. (2014), while the maximum is 69.8%, reported in Bajaj et al. (2023). Similarly, regarding specificity, a significant range is observed, with a minimum of 23.4% in Bajaj et al. (2023) and a maximum of 77.3% in Dominguez, Grotegut, et al. (2018).

The diversity of results highlights significant heterogeneity among the included studies. This heterogeneity may be attributed to various factors, such as differences in participant inclusion criteria, ESS assessment methodologies, and demographic characteristics of the studied populations. Furthermore, the analysis of equality tests reveals statistically significant differences in both sensitivity and specificity among the studies.

Regarding DOR and LR, the values show considerable variation among the studies, with confidence intervals reflecting this diversity. Particularly, the minimum and maximum values of DOR, PosLR, and NegLR provide an overview of the range of diagnostic performances observed among the studies. The test for equality of sensitivities reveals significant differences among the studies (*χ* ^2^ = 10.5501, df = 4, *p* = 0.0321). Similarly, the test for equality of specificities shows statistically significant differences among the studies (*χ* ^2^ = 74.1827, df = 4, *p* = 2.97e−15). The correlation analysis demonstrates a positive correlation between sensitivity and false positive rates, with a correlation coefficient ranging from 0.092 to 0.994 (95% CI), indicating that tests with higher sensitivity tend to have higher false positive rates (Figure 3).

## 4 Discussion

### 4.1 Summary of the Result

Our meta‐analysis and updated systematic review evaluated the diagnostic accuracy of screening questionnaires for OSA in the pregnant population. We confirm that traditional screening tools for OSAS, the Berlin questionnaire and Epworth sleepiness scale, validated by several studies in non‐pregnant populations, present a suboptimal performance in the pregnant group. Our findings evidenced moderate to low performance for these tools in pregnant cohorts. These tools are presumably impacted by physiological changes occurring in pregnancy that could mask or alter the OSA's symptoms (Robertson et al. 2019; Morong et al. 2014). These symptoms are often exacerbated by increased fatigue in pregnancy and frequently disturbed sleep, making self‐reports of fatigue, explored in most questionnaires for OSA, inconsistent and resulting in a high rate of false positives (Antony et al. 2014). These results are compatible with current research on OSA and screening tests. A recent meta‐analysis by Tantrakul et al. evidenced the poor diagnostic accuracy of traditional tools in a population of pregnant women (Tantrakul et al. 2017). Our findings confirm and extend the observations of Tantrakul et al.'s analysis, including two additional studies for ESS and one study for the Berlin questionnaire (Tantrakul et al. 2017). Lockhart et al. also reported suboptimal performance of the screening tools for OSA in the third trimester of pregnancy (Lockhart et al. 2015). Similarly, the studies by Wilson et al. and Facco et al. reached the same conclusions and developed a screening tool, which is specific for pregnancy (Wilson et al. 2013). In their study, the conventional screening tools cannot predict sleep apnoea in high‐risk pregnant women. They suggest the use of newly modified tools that take into account factors such as common snoring and hypertension, which have proved more predictive of OSA during pregnancy (Wilson et al. 2013). Fung et al. examined the possible effects of maternal OSA on foetal growth and postulated that OSA may be associated with lower late‐pregnancy foetal growth (Fung et al. 2013). Olivarez et al. reported that the OSA questionnaire had low sensitivity and specificity (Olivarez et al. 2010). This study had an adequate study design and analysed the efficacy of the screening tools within a sample of the pregnant population (Olivarez et al. 2010). Dominguez et al. examine the prevalence and screening for extremely obese pregnant patients (Dominguez, Grotegut, et al. 2018). They reported a high prevalence of OSA in this group and that the questionnaire used, such as the STOP‐BANG questionnaire and ASA checklist, among others, showed a poor performance. They suggested that age, body mass index, neck circumference, frequently witnessed apnoeas, and a high likelihood of falling asleep while driving were more predictive of OSA (Dominguez, Grotegut, et al. 2018). Also, Bajaj et al. screened OSA in a high‐risk population (high BMI) (Bajaj et al. 2023). Their results showed that the positive predictive value for both Facco's tool and STOP‐BANG was above 70% for the high BMI and high maternal age population, respectively (Facco et al. 2014). Different physiological and methodological standpoints could explain the results, which indicated a poor efficacy of conventional screening tools for OSA in pregnant women, such as the Berlin questionnaire and Epworth sleepiness scale. The first of these tools was developed and validated in a non‐pregnant population and adopted by middle‐aged men (Chiu et al. 2017). The physical characteristics of the pregnant groups differ from those of the non‐pregnant groups, including different fat deposit patterns and hormonal influences on muscle tone, fluid retention, and vascular change (Widen and Gallagher 2014; La Verde et al. 2023). These could contribute to the reduced diagnostic accuracy of these tools in a pregnant population. Some traditional tools used for screening OSA depend on daytime sleepiness symptoms (Bernhardt et al. 2022). Most women reported an increased somnolence during pregnancy, which could represent a pregnancy's physiological modification (Pien et al. 2005). This may cause an overlap of these symptoms and reduce the specificity of tools such as the ESS when applied to the screening of OSA among pregnant women (Bourjeily et al. 2017). In addition, mucosal oedema, increased nasal resistance, and increased oestrogen levels during pregnancy may worsen snoring (Venkata and Venkateshiah 2009). Snoring may not always represent an OSA symptom in a pregnant woman (Sarberg et al. 2014). At least, the existing tools did not consider the impact of gestational weight gain, increased blood volume, and increased heart rate on the OSA questionnaires. These factors, which are pregnancy‐related, may modify the way OSA clinically onsets. This could potentially lead to the underdiagnosis of OSA in pregnant women, highlighting the urgent need for more accurate screening tools.

### 4.2 Strengths and Limitations

This review and meta‐analysis investigate the diagnostic accuracy of screening questionnaires for OSA in pregnancy and included the earlier studies on this topic. Our analysis included 10,043 patients; this represents a strength of this study. Pregnant women represent a singular group with physiological changes related to pregnancy that may affect OSA symptoms and risks, most of the time being ignored in broader OSA research. In addition, OSA symptoms during pregnancy may be more pronounced, and their impact on the respiratory tract and, consequently, on the quality of sleep may exacerbate other diseases like temporomandibular disorder (Minervini, Franco, Marrapodi, Almeida, et al. 2023; Minervini, Franco, Marrapodi, Di Blasio, et al. 2023a, b; Minervini, Franco, Marrapodi, Fiorillo, et al. 2023a, b). However, this study presented different limitations. The main limitations of this review relate to the quality and heterogeneity of the included studies regarding the different study designs, demographic profiles, and methodological differences between studies included in the review, which influence the estimates' comparability and introduce bias. For example, outcome variations might reflect differences in the use of the available screening tools or diagnostic criteria for OSA, making interpretation of the pooled results difficult. Finally, the inclusion of non‐homogeneous studies may influence the generalisability of our findings. The result will likely be influenced by factors that differ between studies, including geographic location, study design, and the prevalence of risk factors such as severe obesity.

### 4.3 Implications

This meta‐analysis has different implications for clinical practice and research. We evidenced the limitations of traditional OSA screening tools in the obstetric population. Our findings reveal a clear reduction in diagnostic precision when applied during pregnancy; this evidenced the need to develop and validate new specific pregnancy questionnaires. Physicians should use precautions when applying standard OSA diagnostic tools among pregnant women, and alternative means or adapted criteria should be used because of the manifestation of pregnancy‐specific symptoms and risk factors. Researchers should evaluate all the pregnancy variables, such as increased blood volume, changes in hormonal levels, and patterns of weight gain, that are known to affect sleep patterns and may potentially worsen or even mask the symptoms of OSA. Moreover, public health policies did not include specific strategies to improve sleep disorders screening and related prenatal care (Young et al. 2002). This underscores the necessity for a good screening and shows the need for increased preventive care measures and better therapeutic strategies. The potential benefits are significant, as untreated OSA may be associated with potential outcomes regarding maternal and neonatal health (Bourjeily et al. 2017, 2020; Luciano et al. 2022; Louis et al. 2012; Chen et al. 2012). Besides the different performance of the screening tools among studies included in the metanalysis, it is imperative to adopt a uniform protocol in OSA research among the pregnant population to understand OSA in pregnancy and enhance the quality of prenatal care. Future research should develop pregnancy‐specific screening tools, with a performance evaluated longitudinally across trimesters.

## 5 Conclusion

This meta‐analysis evidenced the limitations of screening tools for OSA, such as the Berlin questionnaire and Epworth sleepiness scale, when applied to pregnant women and underscored the importance of developing tailored screening tools. These tools should incorporate some of the pregnancy‐related factors. We also show the gap in our understanding and correct management of OSA in pregnant women. Untreated OSA has potentially adverse consequences for the mother and foetal well‐being. Future studies should proceed to develop and validate new screening protocols for OSA during pregnancy. These studies should be preceded by studies that explore pregnancy‐related factors and changes. Longitudinal studies should also be carried out to understand how risks and manifestations of OSA change across different trimesters of pregnancy, thus leading to more dynamic stage‐specific approaches for screening.

## Author Contributions

**Marco La Verde:** conceptualization, data curation, formal analysis. **Esposito Renata:** conceptualization, data curation, methodology, investigation. **Maria Maddalena Marrapodi:** methodology, data curation, writing – original draft. **Luigi Della Corte:** visualization, methodology, software, validation. **Mario Fordellone:** formal analysis, writing – original draft, investigation. **Hande Uzunçıbuk:** writing – original draft, visualization, software. **Vincenzo Ronsivalle:** visualization, writing – original draft, investigation. **Gabriele Cervino:** conceptualization, writing – review and editing, supervision. **Giuseppe Minervini:** conceptualization, supervision, writing – review and editing.

## Conflicts of Interest

The authors declare no conflicts of interest.

## Supporting information


**Appendix 1:** PRISMA 2009 checklist.

**Appendix 2:** Detailed search strategy for systematic review.

**Appendix 3:** Risk of bias assessment across included studies.

## Data Availability

The data that support the findings of this study are available on request from the corresponding author. The data are not publicly available due to privacy or ethical restrictions.
