# Supplementary material for: Diagnostic Accuracy of the Screening Questionnaires for Obstructive Sleep Apnoea in Pregnancy: A Meta‐Analysis and Updated Systematic Review
Source: J Sleep Res. 2025 Sep 25;35(3):e70197. doi: 10.1111/jsr.70197 (PMC13193479; doi:10.1111/jsr.70197)
Supplement: Supplementary file 2 — Appendix 2: Detailed search strategy for systematic review. [file JSR-35-e70197-s002.docx]

**APPENDIX 2**

**Detailed search strategy for systematic review.**

| **Variable** | **Search strategy** |
| --- | --- |
| Database searched | MEDLINE, Scopus, Cochrane Central Register of Controlled Trials (CENTRAL), Google scholar, from their origin until March 11, 2024 |
| Search strategy for Pubmed | “pregnant women”, “sleep questionnaire”, Berlin, “Epworth sleepiness scale” or ESS, “Pittsburgh sleep quality index”, PSQI, “sleep test”, polysomnography, PSG, Watch-PAT; and obstructive sleep  apnea (MeSH) sleep apnea, obstructive(MeSH), “sleep apnea”, OSA, “sleep disordered breathing”, SDB. |
| Search strategy for Scopus | ("pregnant women" OR pregnancy OR pregnant) AND ("sleep questionnaire" OR "Berlin questionnaire" OR "Epworth sleepiness scale" OR ESS OR "Pittsburgh sleep quality index" OR PSQI) AND ("sleep test" OR polysomnography OR PSG OR "Watch-PAT") AND ("obstructive sleep apnea" OR "sleep apnea" OR "sleep apnea, obstructive" OR OSA OR "sleep disordered breathing" OR SDB) |
| Search strategy for Cochrane Central Register of Controlled Trials (CENTRAL) | ("pregnant women" OR pregnancy OR pregnant) AND ("sleep questionnaire" OR "Berlin questionnaire" OR "Epworth sleepiness scale" OR ESS OR "Pittsburgh sleep quality index" OR PSQI) AND ("sleep test" OR polysomnography OR PSG OR "Watch-PAT") AND ("obstructive sleep apnea" OR "sleep apnea, obstructive" OR "sleep apnea" OR OSA OR "sleep disordered breathing" OR SDB) |
| Google scholar | Pregnant women, sleep questionnaire, sleep apnea, OSA, sleep disordered breathing. |
| Other sources | The reference lists of selected articles and reviews were hand searched to identify any  relevant articles. |
