# Supplementary figures and images for: Diagnostic Accuracy of the Screening Questionnaires for Obstructive Sleep Apnoea in Pregnancy: A Meta‐Analysis and Updated Systematic Review
Source: J Sleep Res. 2025 Sep 25;35(3):e70197. doi: 10.1111/jsr.70197 (PMC13193479; doi:10.1111/jsr.70197)

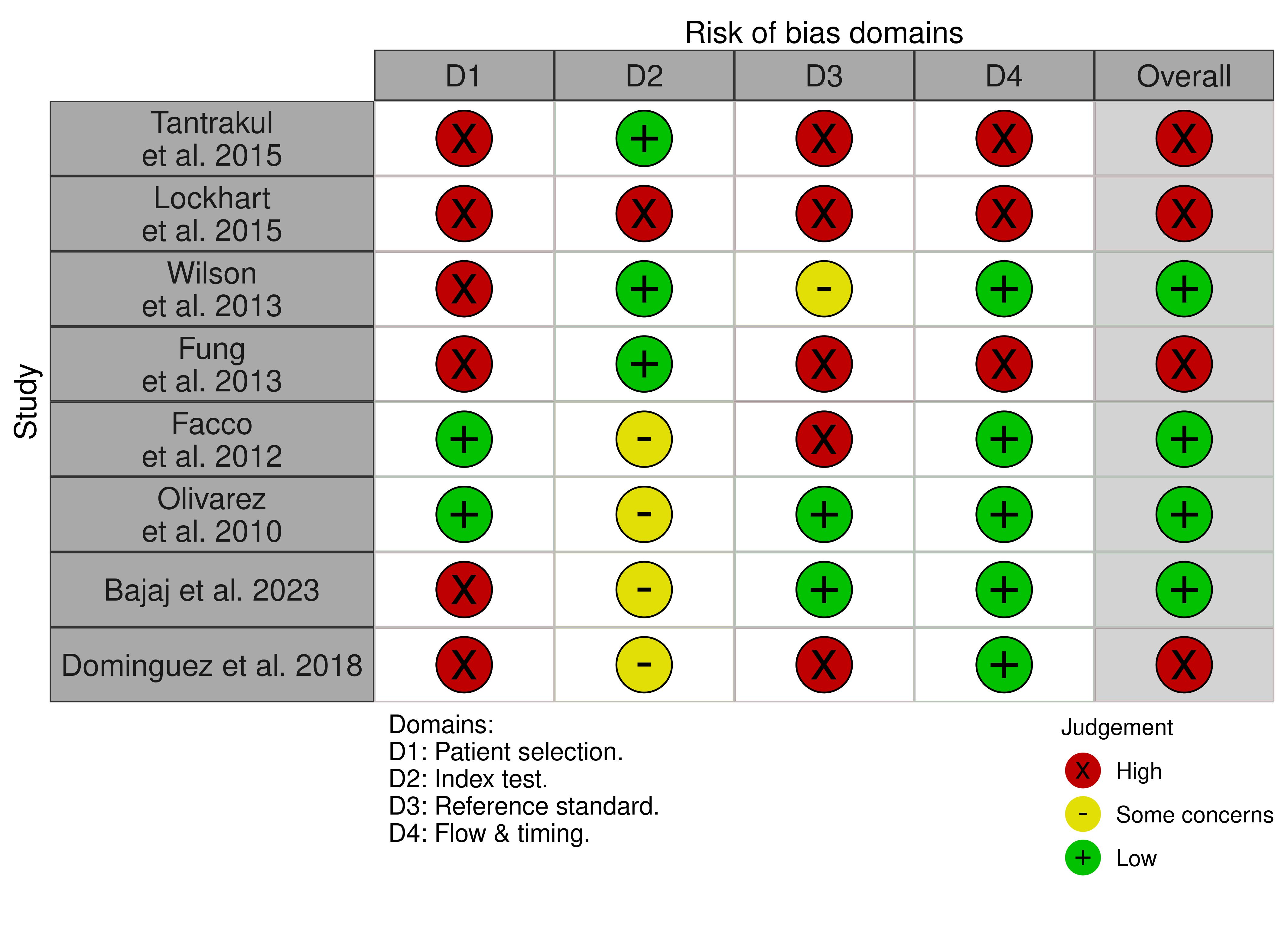

Supplement: Supplementary file 3 — Appendix 3: Risk of bias assessment across included studies. [file JSR-35-e70197-s001.jpeg]
